# Dysfunction in parkin aggravates inflammatory bone erosion by reinforcing osteoclast activity

**DOI:** 10.1186/s13578-023-00973-0

**Published:** 2023-03-07

**Authors:** Eun-Young Kim, Ji-Eun Kim, Young-Eun Kim, Bongkun Choi, Dong Hyun Sohn, Si-On Park, Yeon-Ho Chung, Yongsub Kim, William H. Robinson, Yong-Gil Kim, Eun-Ju Chang

**Affiliations:** 1 Department of Biochemistry and Molecular Biology, Asan Medical Center, University of Ulsan College of Medicine, 88 Olympic-ro 43-gil, Songpa-gu, Seoul, 05505 Korea; 2 Stem Cell Immunomodulation Research Center, University of Ulsan College of Medicine, 88 Olympic-ro 43-gil, Songpa-gu, Seoul, 05505 Korea; 3 Department of Rheumatology, Asan Medical Center, University of Ulsan College of Medicine, 88 Olympic-ro 43-gil, Songpa-gu, Seoul, 05505 Korea; 4 Department of Microbiology and Immunology, Pusan National University School of Medicine, Yangsan, 50612 Korea; 5 Department of Biomedical Sciences, Asan Medical Center, University of Ulsan College of Medicine, 88 Olympic-ro 43-gil, Songpa-gu, Seoul, 05505 Korea; 6 Division of Immunology and Rheumatology, Department of Medicine, Stanford University School of Medicine, Stanford, CA 94305 USA

**Keywords:** Parkin, Osteoclast, Inflammatory bone erosion, Interleukin-1-beta, Acetylated α-tubulin

## Abstract

**Background:**

Parkin dysfunction associated with the progression of parkinsonism contributes to a progressive systemic skeletal disease characterized by low bone mineral density. However, the role of parkin in bone remodeling has not yet been elucidated in detail.

**Result:**

We observed that decreased parkin in monocytes is linked to osteoclastic bone-resorbing activity. siRNA-mediated knockdown of parkin significantly enhanced the bone-resorbing activity of osteoclasts (OCs) on dentin without any changes in osteoblast differentiation. Moreover, *Parkin*-deficient mice exhibited an osteoporotic phenotype with a lower bone volume accompanied by increased OC-mediated bone-resorbing capacity displaying increased acetylation of α-tubulin compared to wild-type (WT) mice. Notably, compared to WT mice, the *Parkin*-deficient mice displayed increased susceptibility to inflammatory arthritis, reflected by a higher arthritis score and a marked bone loss after arthritis induction using K/BxN serum transfer, but not ovariectomy-induced bone loss. Intriguingly, parkin colocalized with microtubules and parkin-depleted-osteoclast precursor cells (*Parkin*^−/−^ OCPs) displayed augmented ERK-dependent acetylation of α-tubulin due to failure of interaction with histone deacetylase 6 (HDAC6), which was promoted by IL-1β signaling. The ectopic expression of parkin in *Parkin*^−/−^ OCPs limited the increase in dentin resorption induced by IL-1β, accompanied by the reduced acetylation of α-tubulin and diminished cathepsin K activity.

**Conclusion:**

These results indicate that a deficiency in the function of parkin caused by a decrease in parkin expression in OCPs under the inflammatory condition may enhance inflammatory bone erosion by altering microtubule dynamics to maintain OC activity.

**Supplementary Information:**

The online version contains supplementary material available at 10.1186/s13578-023-00973-0.

## Background

Parkinson’s disease (PD) is a neurodegenerative disorder characterized by involuntary shaking and muscle rigidity. Due to the motor imbalance, patients with PD are at a high risk of falling and immobility, leading to increased incidences of fractures [1–3]. Conversely, accumulating evidence indicates a reduced bone mineral density (BMD) and a high percentage of osteopenia in patients with PD [4, 5]. In addition, an increased risk for osteoporosis is reported in PD cases [6, 7], indicating a relationship between PD and the pathological change in bone remodeling during PD progression.

The progression of PD and its pathology is associated with the dysregulation of genes, such as α-synuclein, LRRK2, DJ-1, parkin, and PINK [8]. Among them, parkin is an E3 ubiquitin ligase that is the most prevalent genetic factor in familial PD, and its mutations account for up to 50% of recessive PD cases [9–11]. It is well established that parkin has a protective role against cellular stress [12] and also functions in cell cycle regulation [13]. Mutations in parkin lead to mitochondrial dysfunction and enhanced oxidative stress in neuronal cells and could contribute to the pathogenesis of PD [14, 15]. Besides neuronal cell function, parkin is also involved in a wide array of biological processes, such as differentiation, as well as the regulation of lipid metabolism, innate immunity against infection, and tumorigenesis [16–18]. However, its role in bone metabolism is not yet defined in any detail.

The immune response is another pathology of PD in which there is a burden of cytokines, chemokines, and inflammatory cells in both the central and peripheral immune systems [19]. Interleukin-1-beta (IL-1β) has a multi-faceted character in promoting the pathogenesis of PD [20, 21]. This proinflammatory cytokine is identified as an osteoclast (OC) activating factor that potentiates OC function [22, 23]. Upon IL-1β stimulation, extracellular signal-regulated kinase (ERK) signaling is activated in the receptor activator of nuclear factor kappa-B ligand (RANKL)-induced OCs [24], which stimulates OC formation by activating the proliferation of OC precursors [25] and maintains the survival of OCs for bone resorption [26], provoking an excess of bone destruction under pathological conditions, such as in inflammatory bone diseases [27–29]. For instance, IL-1β can induce bone erosion at the joint inflammation sites of rheumatoid arthritis (RA) via the orchestrated activation of OCs [30, 31] and the induction of other proinflammatory cytokines, such as tumor necrosis factor (TNF) and interleukin-6 (IL-6), which are secreted within inflamed synovium in patients with RA, contributing to the pain and functional disability [27–29]. The clinical evidence that PD causes joint deformities mimicking RA [32, 33] supports the possible link between PD progression and inflammatory bone loss, although direct molecular evidence is currently lacking.

To maintain bone homeostasis, the appropriate bone-resorbing activity of OCs is important. One of the most important factors for regulating the bone-resorbing activity of OCs is the formation of the podosome belt, a distinct arrangement of actin-rich adhesion structures called podosomes in OCs that requires a microtubule network [34, 35]. The acetylation of tubulin stabilizes the OC microtubule system, which culminates in increased bone-resorbing activity. According to Destaing et al. [36], the microtubule acetylation is, in turn, controlled by the Rho (a small GTPase of the Ras superfamily)-diaphanous-related formin (mDia2)-histone deacetylase 6 (HDAC6) pathway, indicating the essential role of HDAC6 in the regulation of tubulin deacetylation. This is further supported by the loss of the podosome belt upon disruption of the microtubule network, which enhances the secretion of cathepsin K, increasing the bone matrix resorbing activity of OC [37, 38]. Numerous studies show that parkin modulates the stability of microtubules [39–41], supporting that parkin may serve to regulate OC function. To date, however, there has been no direct evidence for the involvement of those genes in bone metabolism. Here, we report that *Parkin*-deficient mice display a reduced bone mass accompanied by increased OC activity via the regulation of α-tubulin acetylation and are susceptible to bone loss under an inflammatory condition. These results reveal parkin as a potential determinant of the maintenance of bone turnover balance in PD by regulating microtubule dynamics required for OC function.

## Results

### Parkin silencing promotes OC activity in vitro

**Fig. 1:**
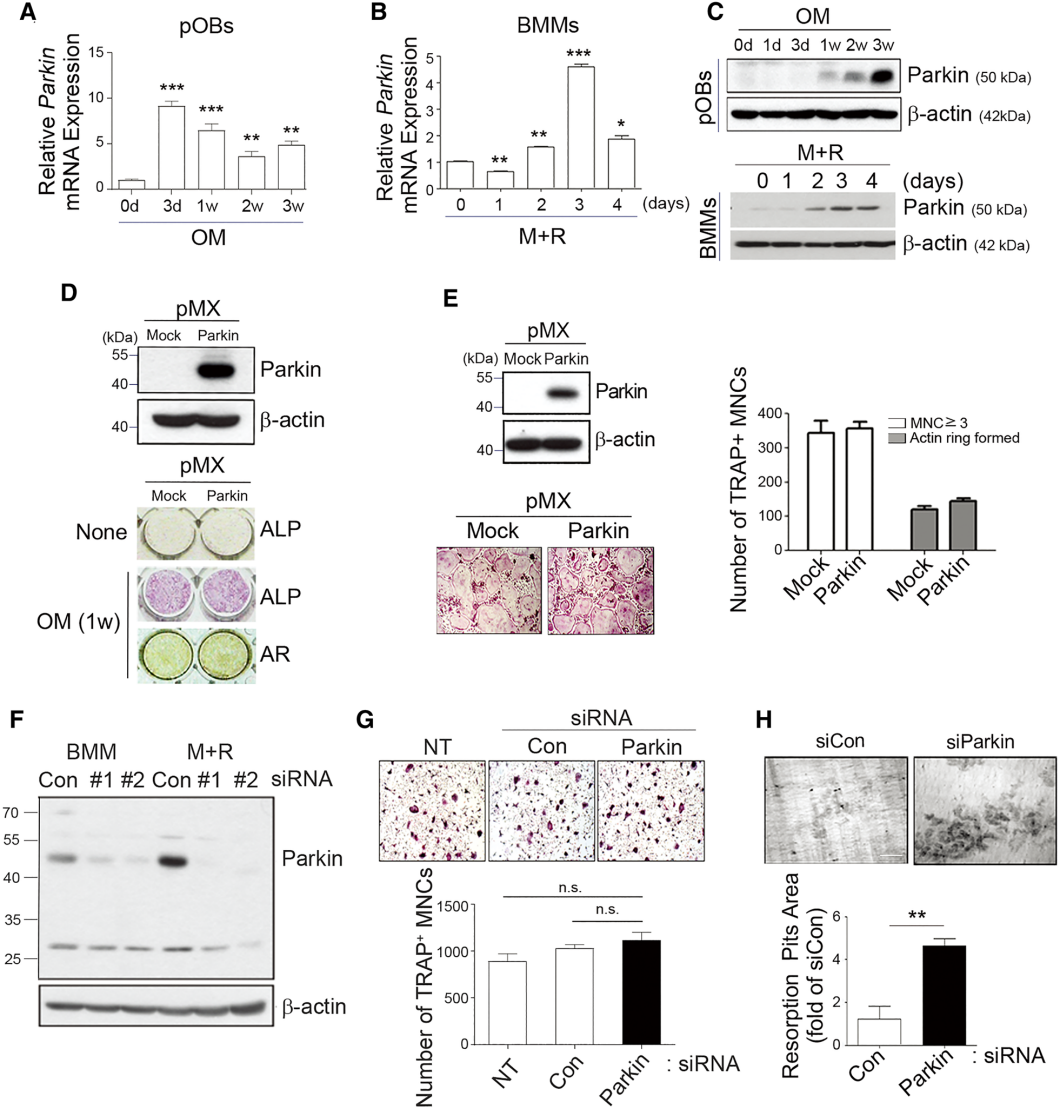
Silencing of parkin enhanced bone-resorbing activity. **A**–**C** Parkin mRNA and protein levels were determined by **A** qPCR and **C** (upper) western blot analysis, respectively, during differentiation of OBs in OM at the indicated times. BMMs were cultured with M-CSF (M) and RANKL (R) to induce differentiation into mOCs. Parkin transcripts and protein levels were determined by **B** qPCR and **C** (lower) western blot analysis. **D** Overexpression of parkin in primary mouse calvarial osteoblastic precursor cells generated by a virus infection system was detected by western blot analysis (upper). After 1 week, the cells were stained with ALP and AR to assess the degree of OB differentiation (lower). **E** Overexpression of parkin in OCPs using a virus infection system was confirmed by western blot analysis (left, upper). The infected cells were stained with TRAP solution (left, lower), and TRAP-positive MNCs were counted under a light microscope (×100) (right). **F** BMMs or OCPs primed with M-CSF (M) and RANKL (R) were transfected with control (Con) or parkin-specific siRNA #1 and #2 and incubated with fresh medium or osteoclastogenic medium for 24 h. The cell lysates were collected and analyzed by western blot analysis to quantify the levels of parkin protein expression. **G** Transfected cells were additionally cultured for 3 days in osteoclastogenic medium and stained with TRAP solution (upper). TRAP-positive MNCs (≥ 3 nuclei) were counted (lower) under a light microscope (×100). n.s. indicates not significant. **H** RANKL-primed OCPs on dentin were transfected with Con or parkin-specific siRNA. Resorption pits were visualized by staining with toluidine blue (upper), and the resorption pit area was quantified (lower). Original magnification, ×200. Scale bar, 100 μm. *P < 0.05, **P < 0.005, and ***P < 0.001 between the indicated groups. Data are represented as means ± SD from three independent experiments; *P*-values were calculated by Kruskal–Wallis or Tukey post hoc comparison tests

We sought to determine whether parkin plays a potential role in the differentiation of osteoblasts (OBs) or OCs by measuring its expression in these cells by quantitative PCR (qPCR) and western blot analysis. As shown in Fig. 1A–C, the mRNA and protein levels of parkin gradually increased during OB or OC differentiation. However, in early differentiating OBs or OCs, transfection with a retrovirus harboring the parkin gene did not affect their differentiation, as revealed by alizarin red S (AR) staining and alkaline phosphatase (ALP) activity for OBs and tartrate-resistant acid phosphatase (TRAP) staining for OCs (Fig. 1D, E). We then investigated whether parkin deficiency might be involved in bone destruction. Even though there were no significant differences in OC differentiation (Fig. 1F, G), the siRNA-mediated silencing of parkin in OCs induced robust bone resorption compared to a control siRNA (Fig. 1H), suggesting that a reduced expression of parkin may be linked to the bone pathology.

### Parkin deficiency promotes OC activity and decreases bone mass in vivo

**Fig. 2:**
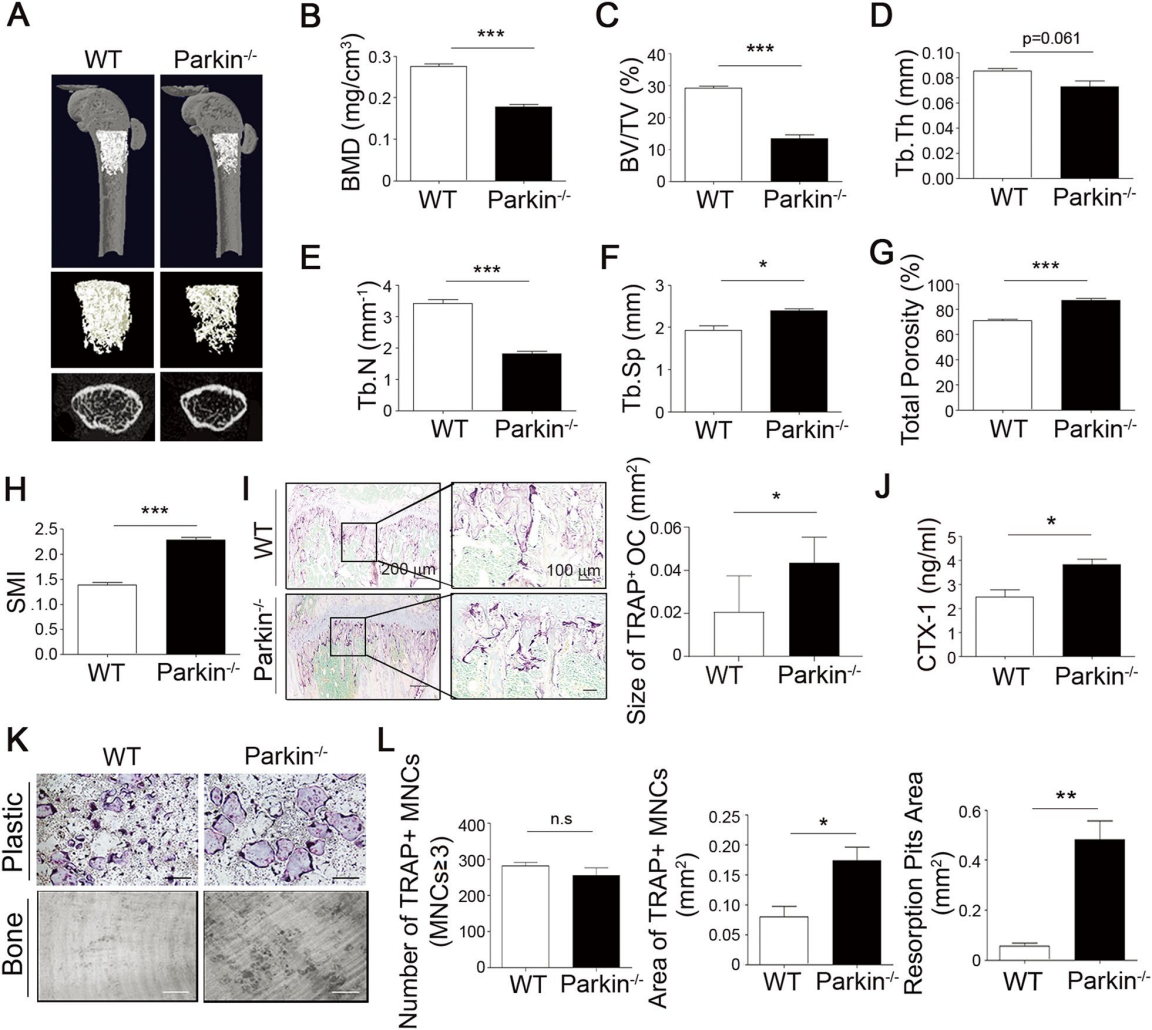
Parkin deficiency enhances the bone-resorption capacity leading to bone loss in vivo. **A** Femurs from WT (n = 5) and *Parkin*^−/−^ (n = 5) male mice were isolated at 8 weeks of age, fixed in 4% paraformaldehyde, and then examined by micro-CT imaging. Representative micro-CT images of the trabecular bone of the mouse femurs are shown. The histograms represent the three-dimensional structural parameters of the femurs. **B**–**H** Three-dimensional morphometric analysis of bone parameters (i.e., **B** bone mineral density; BMD); **C** bone volume per tissue volume (BV/TV), **D** trabecular thickness (Tb. Th), **E** trabecular number (Tb. N), **F** trabecular separation (Tb. Sp), **G** total porosity (%), and **H** structure model index (SMI). **I** Representative TRAP staining images from the femurs of 8-week-old WT and *Parkin*^−/−^ mice are shown (left), and the size of the TRAP^+^ OCs was measured using ImageJ software (right). Scale bar, 200 μm (left) and 100 μm (right). **J** CTX-1 protein levels in the plasma were determined by ELISA. **K**, **L** BMMs from WT or *Parkin*^−/−^ mice were **K** (upper) treated with RANKL for 3 days, fixed, and TRAP-stained, and also **K** (lower) plated onto bone slices and incubated with M-CSF and RANKL for 7 days. Scale bar, 100 μm. **L** (left) The numbers of TRAP-positive multinucleated cells (≥ 3 nuclei) were quantified under a light microscope. **L** (middle) The area of TRAP-positive OCs was evaluated by ImageJ software. Resorption pits were visualized by **K** (lower) staining with toluene blue, and **L** (right) resorption pit areas were then estimated. *P < 0.05, **P < 0.005, and ***P < 0.001 between the indicated groups. Data are represented as means ± SD from three independent experiments; *P*-values were calculated by Kruskal–Wallis or Tukey post hoc comparison tests

Next, we compared the bone phenotypes of WT and *Parkin*-deficient (*Parkin*^−/−^) mice to elucidate the bone-related function of parkin in vivo. A three-dimensional visualization of the distal femoral area revealed a significant loss of trabecular bone in *Parkin*^−/−^ mice (Fig. 2A). The BMD levels of *Parkin*^−/−^ mice were markedly reduced by 34% in comparison to WT mice (Fig. 2B). *Parkin*^−/−^ mice also exhibited a lower bone volume per tissue volume (BV/TV), trabecular thickness (Tb. Th), and trabecular number (Tb. N) (Fig. 2C–E). By contrast, bone-resorbing parameters, such as trabecular separation (Tb. Sp), bone porosity, and the structure model index (SMI), tended to be higher in *Parkin*^−/−^ mice (Fig. 2F–H), demonstrating spontaneous bone loss in these mice. We found larger OCs in the femoral bones of *Parkin*^−/−^ mice compared to WT mice (Fig. 2I). In addition, we observed that the expression of serum-bone resorption (OC) marker, C-telopeptide of collagen type 1 (CTX-1), in the plasma of *Parkin*^−/−^ mice was significantly higher than that of the control WT mice (Fig. 2J). Thus, we observed that *Parkin*-deficient mice display augmented OC activity and reduced bone mass. Moreover, we assessed the involvement of parkin in OC formation and function by in vitro culturing of bone marrow cells from *Parkin*^−/−^ and WT mice with the OC-inducing cytokines macrophage colony-stimulating factor (M-CSF) and RANKL. Although the number of TRAP-positive OCs (MNCs ≥ 3 nuclei) exhibited no significant difference (Fig. 2L, left), the area of TRAP-positive MNCs (Fig. 2K, upper and Fig. 2L, middle) was potently augmented in *Parkin*^−/−^ OCs compared with WT OCs. In particular, the area of resorption pits on the dentin slices was significantly higher in *Parkin*^−/−^ OC cultures than in WT cultures (Fig. 2K, lower and Fig. 2L, right), further supporting the notion that a parkin deficiency leads to a noticeable increase in bone-resorbing activity.

### Parkin deficiency enhances the acetylation of α-tubulin dependently of RANKL-dependent ERK activation in OCs

**Fig. 3:**
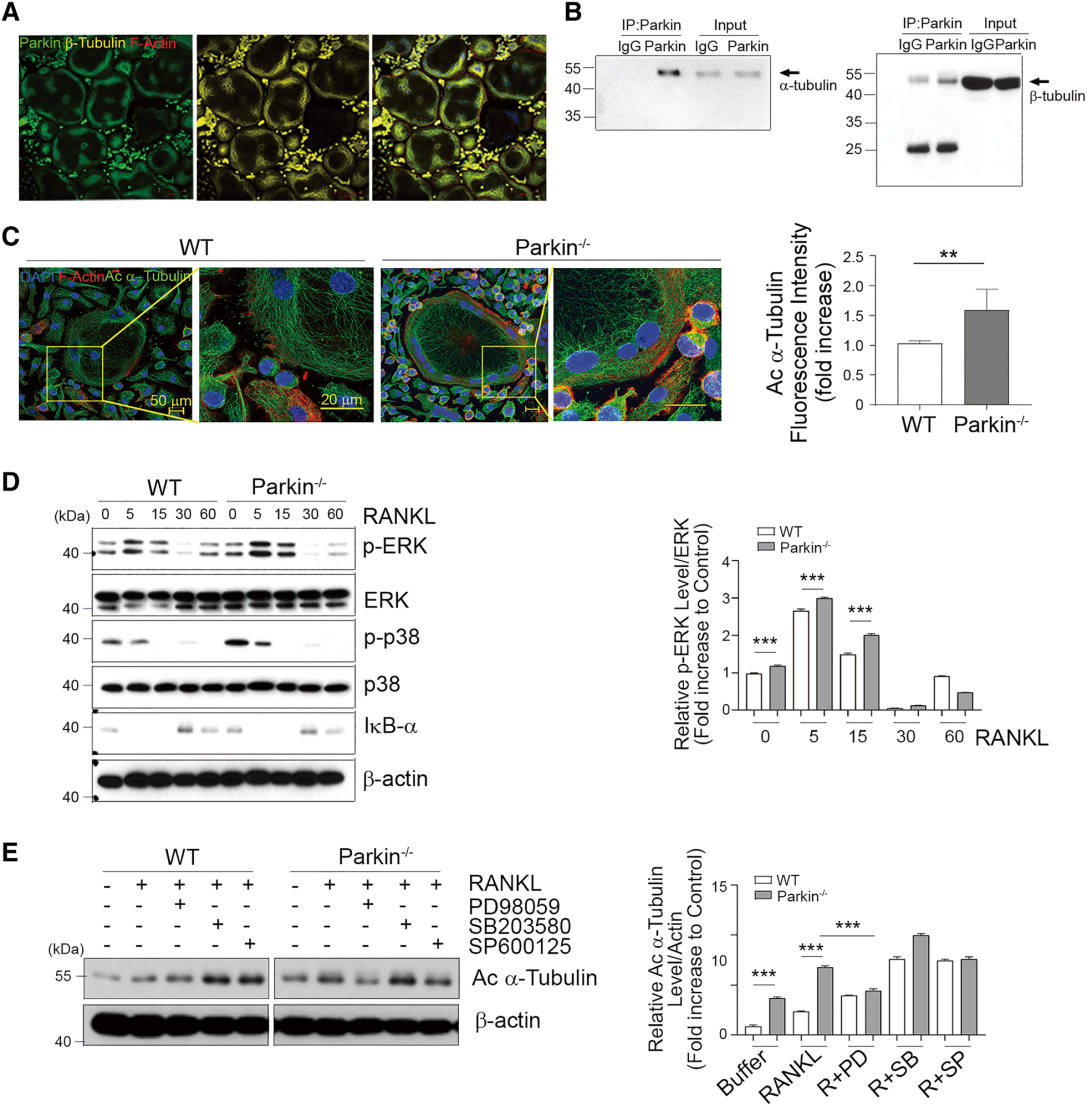
Parkin attenuates the acetylation of tubulin during osteoclast formation through the RANKL-dependent ERK pathway. **A** Mature osteoclasts (mOCs) from WT mice grown on glass coverslips were stained with Cy™3-conjugated antibodies (for β-tubulin; yellow) and FITC-conjugated antibodies (for parkin; green), counterstained for nuclei (DAPI, blue), and observed by confocal microscopy. **B** Cell lysates from mOCs were immunoprecipitated with an antibody recognizing parkin. Precipitated proteins and a 1% protein lysate input were analyzed using western blot analysis with anti-α-tubulin or -β-tubulin antibodies. Experiments were repeated at least three times with similar results. **C** mOCs of WT and *Parkin*^−/−^ mice were grown on glass coverslips and stained using antibody DyLight™ 488-conjugated (for acetylated α-tubulin; green) and antibody DyLight™ 649-conjugated (for F-actin; red), counterstained for nuclei (DAPI, blue), and observed by confocal microscopy (left). Scale bar, 50 μm (left) and 20 μm (right). Fluorescence intensity of acetylated α-tubulin (Ac α-tubulin) was quantified (right). **P < 0.005 between the indicated groups. Data are represented as means ± SD from three independent experiments; *P*-values were calculated by the Kruskal–Wallis test. **D** Pre-fused OCs by M-CSF and RANKL for 2 days from WT and *Parkin*^−/−^ BMMs were starved for 4 h and then retreated with RANKL at the indicated times, and the cells were analyzed by western blot. Representative data from three independent experiments are shown. **E** Pre-fused OCs from WT and *Parkin*^−/−^ mice were starved for 4 h and then treated with RANKL in the presence of buffer, PD98059 (ERK inhibitor), SB203580 (p38 inhibitor), or SP600125 (JNK inhibitor). After 24 h, the cells were harvested and analyzed by western blot (upper), and their acetylated tubulin level was quantified (right). Data are represented as means ± SD from three independent experiments. **P < 0.005 and ***P < 0.001 vs. the WT group. *P*-values were calculated by Tukey post hoc comparison tests

As mentioned above, microtubules play a critical role in the expansion of podosomes into the belt during OC maturation for bone resorption [34], and those microtubules are regulated by phosphorylation and acetylation events [42]. In addition, parkin has been shown to interact with tubulin and, in turn, stabilize microtubules [43]. To investigate the regulatory role of parkin in controlling OC activity by interacting with microtubules, we assessed the localization of parkin in mature OCs. The assessment of parkin by confocal laser scanning microscopy showed that its staining signals were mostly confined to the internal tubulin border area of the OCs and were not evident in the specific actin-rich sealing zone (Fig. 3A). We also observed that the endogenous parkin colocalizes with β-tubulin but not F-actin, as revealed by the immunostaining of mature OCs (Fig. 3A and Additional file 1: Fig. S1). Parkin was further found to be strongly associated with tubulin heterodimers and microtubules (α- and β-tubulin), as evidenced by co-immunoprecipitation of tubulin and parkin using specific parkin antibodies (Fig. 3B). Although parkin interacted with both α- and β-tubulin (Fig. 3B), the binding to α-tubulin appeared to be relatively stronger. Given the literature findings of a close correlation between the stability of the podosome belt and the acetylation of microtubules [34, 38, 42], we next explored the possibility that parkin is involved in the acetylation of tubulin. An enhanced acetylation of α-tubulin was exhibited along the interior zone and also intensively detected around the internal border area of parkin-depleted OCs (Fig. 3C, left). In addition, the acetylation of α-tubulin was markedly augmented in parkin-deficient cells compared with WT cells, and the level of acetylated α-tubulin gradually increased in OCs (Fig. 3C, right). To provide a mechanistic explanation for the increase in the acetylation of α-tubulin by parkin deficiency, we compared the RANKL-induced signaling pathway, including activation of nuclear factor kappa B (NF-κB) and mitogen-activated protein kinases (MAPKs) [44] in bone marrow-derived macrophages (BMMs) isolated from WT or *Parkin*^−/−^ mice (Fig. 3D, E). Western blot analysis revealed an increase in ERK and p38 activation in response to RANKL in BMMs of *Parkin*^−/−^ mice compared to WT mice (Fig. 3D). Thus, we next investigated whether acetylation of α-tubulin could be modulated through the MAPK signaling pathway. To this end, we added various signaling inhibitors in response to RANKL in OCs. We found that the inactivation of phosphorylated ERK (pERK) by PD98059 led to a significant reduction in the acetylated α-tubulin levels in *Parkin*^−/−^ OCs (Fig. 3E) but not in WT OCs, suggesting that parkin might restrain the activity of α-tubulin via ERK. In comparison, p38 and c-Jun N-terminal kinase (JNK) inhibition did not significantly change in the RANKL-induced acetylation of α-tubulin. These observations suggested that the ERK-acetylated tubulin axis may act as a potential component mediator of OC activity downstream of the parkin pathway.

### Parkin deficiency regulates the acetylation of α-tubulin by interacting with HDAC6 in OCs

**Fig. 4:**
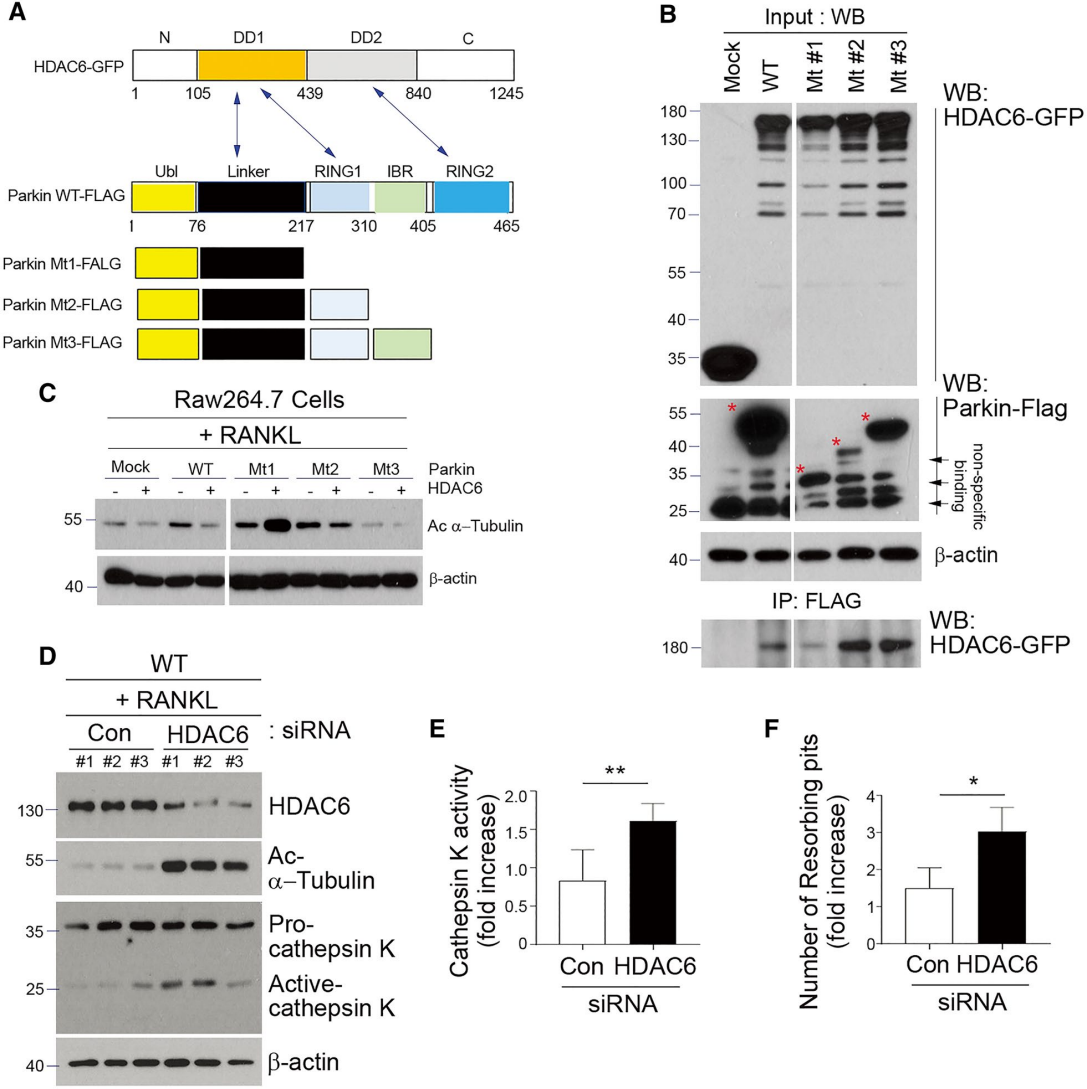
Decrease in acetylation of α-tubulin is modulated by the interaction between parkin and HDAC6. **A**–**C** Schematic representation of full-length parkin protein and its deletion mutants binding to HDAC6. **A** Blue arrows indicated the binding region between parkin and HDAC6. **B** Parkin interacts with HDAC6. Empty vector, FLAG-tagged full-length parkin (1-465), Mt1 (1-217), Mt2 (1-310), or Mt3 (1-405) was co-transfected with GFP-HDAC6 in HEK 293 cells. Anti-FLAG immunoprecipitation was performed, followed by western blotting using anti-GFP antibodies. The input of FLAG for parkin, GFP for HDAC6, or β-actin is also shown. Red asterisk indicated overexpressed mutant form of parkin. **C** Interaction between parkin and HDAC6 is decreased by the Mt1 lacking RING1, IBR, and RING2 of parkin. Empty vector, FLAG-tagged full-length parkin, Mt1, Mt2, or Mt3 was co-transfected with GFP-HDAC6 in RAW 264.7 osteoclast precursor cells and was further cultured for 3 days (including M-CSF and RANKL), followed by western blotting using anti-acetylated α-tubulin, or anti-β-actin. **D**–**F** BMMs from WT mice were transfected with control- or HDAC6-specific siRNA and subsequently cultured with 30 ng/ml M-CSF and 10 ng/ml RANKL for 3 days. **D** Protein expression levels of HDAC6, acetylated α-tubulin, and cathepsin K were analyzed by western blot analysis. **E** Cathepsin K activity and **F** the number of resorption pits from the transfected cells were measured. Data represented as means ± SD from three independent experiments; *P < 0.05 and **P < 0.005 vs. the WT group. *P*-values were calculated by Tukey post hoc comparison tests

Parkin and HDAC6, a known deacetylase, are reported to bind to tubulin α/β heterodimers [45, 46]. Hence, we reasoned that the interaction of parkin with HDAC6 may affect the status of α-tubulin acetylation to regulate microtubule stability during OC activation. To test this possibility, we made mutant proteins with multiple interaction domains of parkin bound to the DD1 and/or DD2 domain of HDAC6 and then expressed parkin or its domains with HDAC6 in HEK 293 cells, as shown in Fig. 4A. The full-length parkin (WT), mutant 2, or mutant 3 of the parkin RING1 domain was binding with HDAC6 as shown in anti-FLAG (parkin) immunoprecipitation, while mutant 1 lacking RING1 of parkin did not interact with HDAC6 (Fig. 4B), indicating that parkin interacts with HDAC6 via its RING1 domain. This interaction was responsible for the deacetylation of α-tubulin in OCs in response to RANKL (Fig. 4C). HDAC6 knockdown substantially increased the acetylation of α-tubulin and was accompanied by the activation of cathepsin K and bone-resorption activity in mature OCs (Fig. 4D–F). Taken together, these results suggested that parkin may primarily regulate the acetylation of α-tubulin through its interaction with HDAC6 to exert its influence on the bone-resorbing capacity of OCs.

### Parkin deficiency increases the susceptibility to inflammatory arthritis but not ovariectomy (OVX)-induced bone loss

**Fig. 5:**
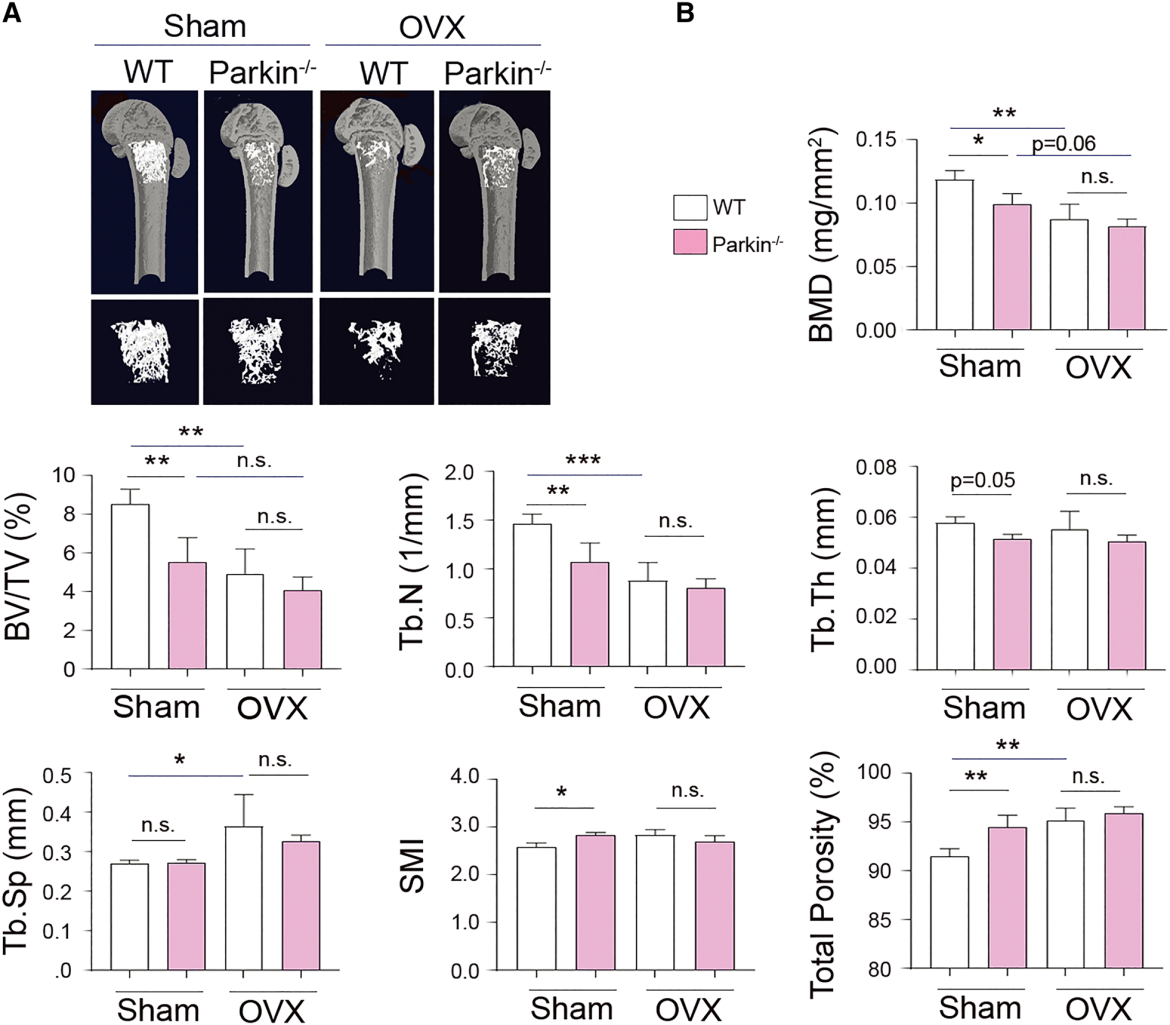
Parkin deficiency is not involved in OVX-induced bone loss. **A**, **B** Femurs from WT (n = 5, each) and *Parkin*^−/−^ (n = 5, each) female mice-subjected to sham or OVX were isolated, fixed in 4% paraformaldehyde, and then examined by micro-CT imaging. **A** Representative images of femoral bones of different groups. **B** The histograms represent three-dimensional structural parameters of the femurs: bone mineral density (BMD), bone volume per tissue volume (BV/TV), trabecular thickness (Tb. Th), trabecular number (Tb. N), trabecular separation (Tb. Sp), total porosity (%), or structure model index (SMI). Statistical analysis involved ANOVA, followed by the Kruskal–Wallis test using Prism version 3.0 software. *P < 0.05, **P < 0.005, or ***P < 0.001 compared with indicated group, n.s. indicates not significant

**Fig. 6:**
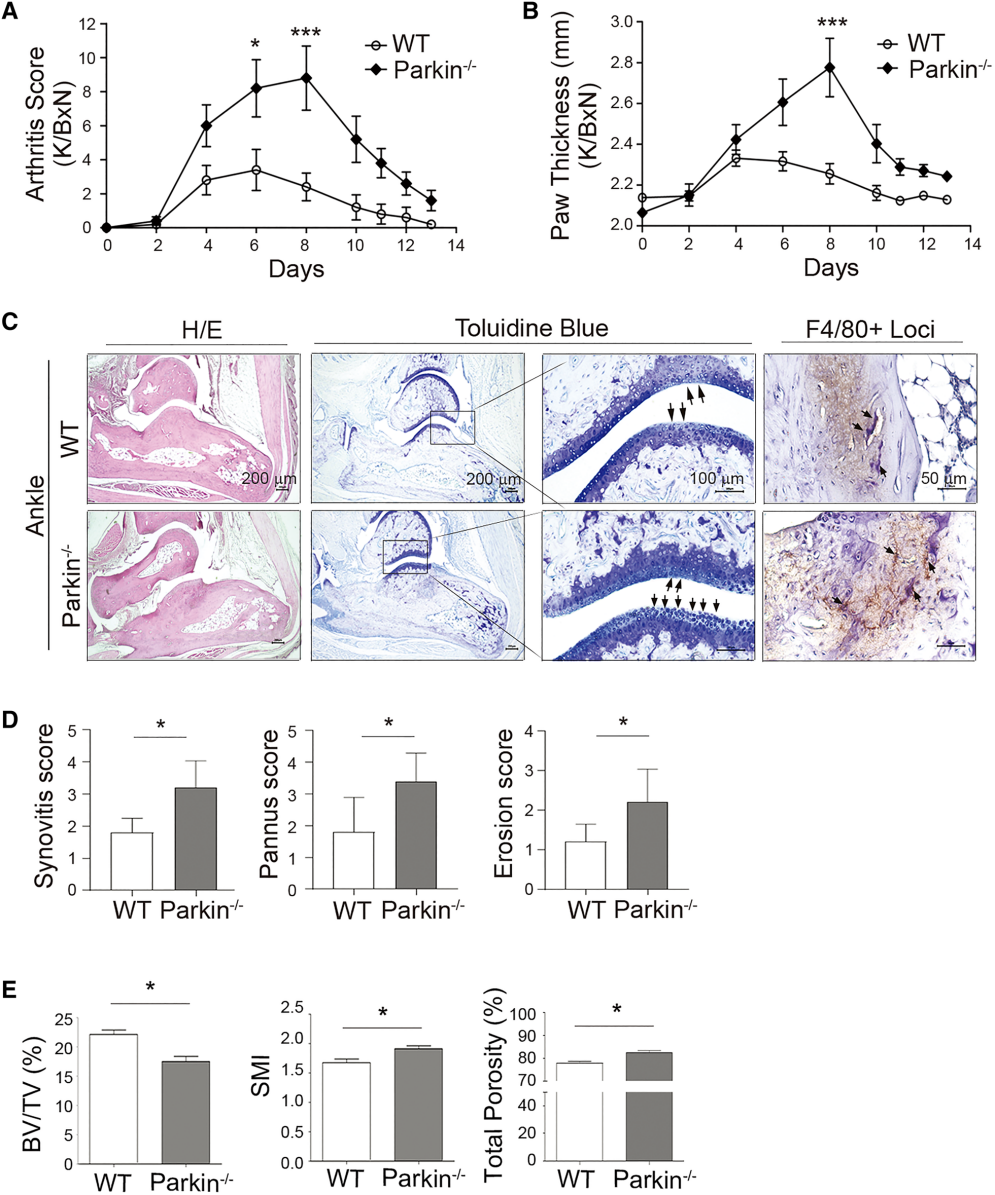
Effects of parkin deficiency of inflammatory bone erosion in a K/BxN serum-transfer arthritis mouse model. **A**, **B** Adult K/BxN mice were bled, and the sera were pooled. WT and *Parkin*^−/−^ mice were intraperitoneally injected on days 0 and 2 with 200 µl of pooled K/BxN. Arthritis was **A** clinically scored, and **B** paw thickness was measured with a caliper for up to 13 days. The means ± SD of five mice per group are shown. **C** Ankle joints of K/BxN serum-transfer arthritis model mice were stained with hematoxylin/eosin (H/E) or toluidine blue (blue). Immunohistochemical analysis was performed to detect F4/80 (brown). The arrows indicate bone-destructed loci of ankle joints (middle) or F4/80^+^ macrophages (right), respectively. **D** Synovitis, pannus, and erosion scores were quantified. **E** Parkin deficiency enhances the bone-resorption capacity. Quantitative histomorphometry of the distal femur of K/BxN-induced WT and *Parkin*^−/−^ mice (n = 4–7). Bone volume per tissue volume (BV/TV) (left), structure model index (SMI) (middle), and total porosity (%) (right) values were measured by peripheral quantitative computed tomography. Data represented as means ± SD. *P < 0.05 and ***P < 0.001 between the indicated groups. *P*-values were calculated by Kruskal–Wallis or Tukey post hoc comparison tests

To clarify the role of parkin in bone metabolism, *Parkin*^−/−^ mice underwent an exaggerated state of estrogen deficiency-induced bone remodeling and were compared with WT mice. WT and *Parkin*^−/−^ mice were either OVX or sham-operated and subjected to micro-computed tomography (micro-CT) assessment at 4 weeks post-operation (Fig. 5A). Micro-CT analysis showed that OVX successfully induced a bone loss in WT and *Parkin*^−/−^ mice, but there was no significant increase in ovariectomized *Parkin*^−/−^ mice compared to ovariectomized WT mice (Fig. 5). It was previously reported that lack of parkin increases inflammation induced by IL-1β, which leads to accumulation of damaged mitochondria in human articular chondrocytes [47], implying an association between parkin and IL-1β-associated signaling. In agreement with these observations [48], IL-1β stimulation reduced parkin expression in mouse primary BMMs and mature OCs (Additional file 1: Fig. S2). These findings supported the evidence for the decreased expression of parkin in monocytes under the inflammatory condition. Given that IL-1β is increased in the inflamed joint of the K/BxN-induced arthritis model due to its critical role in inflammatory bone loss [49], we determined whether parkin is involved in the pathogenesis of inflammatory arthritis and bone density using a K/BxN serum-transfer arthritis mouse model. Both WT and *Parkin*^−/−^ mice began to develop arthritis 4 days after immunization with K/BxN (Fig. 6). Nonetheless, with regards to inflammatory arthritis, the increase in the mean arthritis scores (Fig. 6A) and paw thickness (Fig. 6B) of the *Parkin*^−/−^ mice was more profound than that of the WT mice. In addition, synovitis, pannus, and erosion scores were higher in *Parkin*^−/−^ mice than in WT mice in the K/BxN serum-transfer model (Fig. 6C, D). Moreover, bone destruction and inflammatory F4/80-positive macrophages were abundantly observed in *Parkin*^−/−^ mice (Fig. 6C). Concomitantly, a three-dimensional visualization of the distal femur in these animals indicated a massive loss of trabecular bone in K/BxN *Parkin*^−/−^ mice (Fig. 6E). In addition, analysis of the micro-CT data revealed a markedly reduced BV/TV (%) in K/BxN *Parkin*^−/−^ mice (Fig. 6E, left), whereas the SMI and total bone porosity were significantly increased in K/BxN *Parkin*^−/−^ mice compared with K/BxN WT mice (Fig. 6E, middle and right). Collectively, these results suggested that parkin may protect against bone destruction mediated by inflammatory stimuli.

### Ectopic parkin suppresses IL-1β-mediated OC activity

**Fig. 7:**
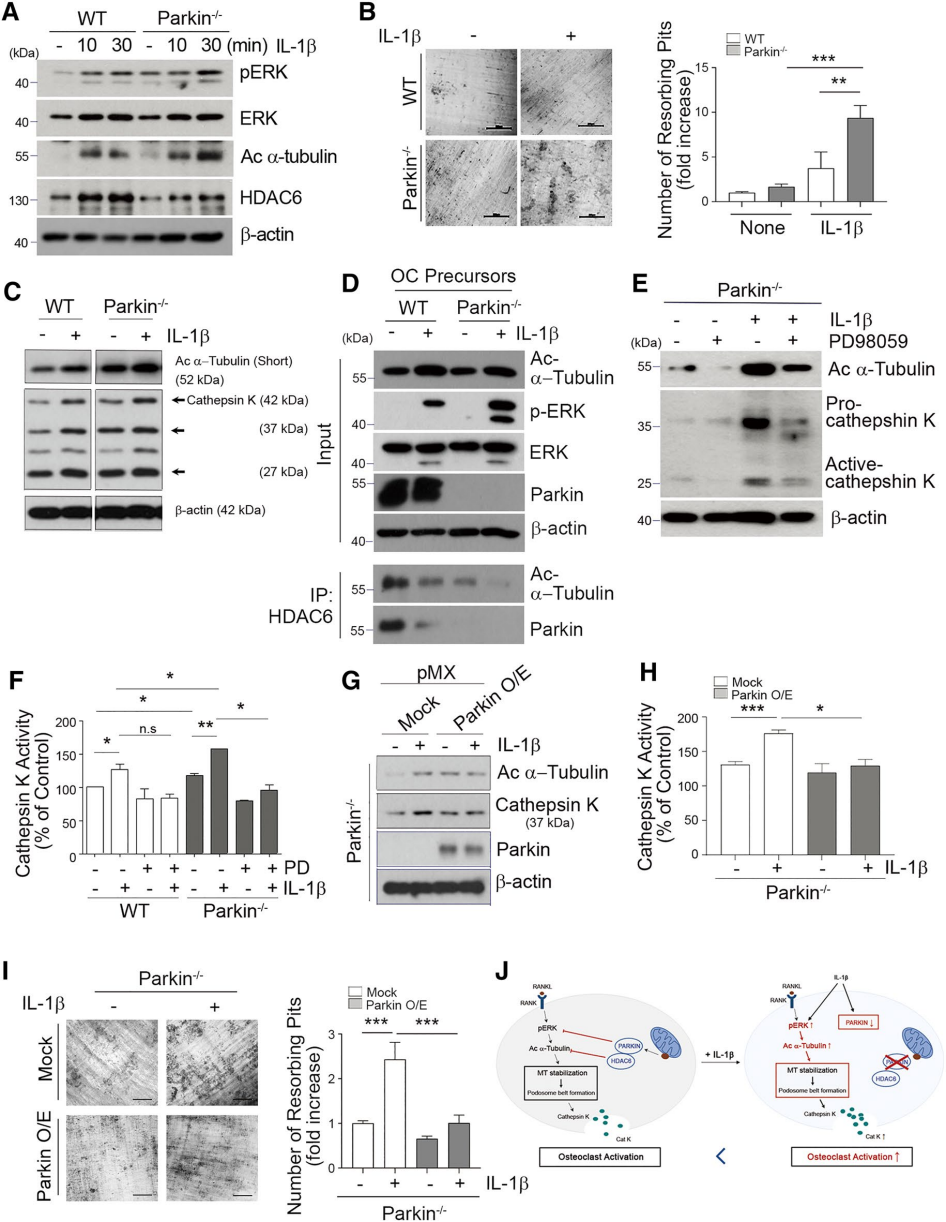
Deficiency of parkin promotes IL-1β-induced OC resorptive capacity. **A** OCPs of WT or *Parkin*^−/−^ mice were treated with IL-1β for 48 h in the presence of RANKL. OC cells were starved for 4 h, retreated with IL-1β, and analyzed for pERK, ERK, acetylated α-tubulin, or HDAC6 expression by western blot. **B** BMMs from WT or *Parkin*^−/−^ mice were plated onto a bone slice and further incubated in the presence of the IL-1β. **C** OCPs of WT or *Parkin*^−/−^ mice were treated with IL-1β in the presence of RANKL and then analyzed for acetylated α-tubulin, cathepsin K, or β-actin expression by western blot. **D** OCPs of WT or *Parkin*^−/−^ mice were cultured with IL-1β. Afterward, anti-HDAC6 immunoprecipitation was performed, followed by western blotting with acetylated α-tubulin or parkin antibodies. The input of acetylated α-tubulin, pERK, ERK, parkin, or β-actin is shown. **E**, **F** RANKL-primed OCPs of *Parkin*^−/−^ were treated with IL-1β with PD98059. **E** Cells were harvested and analyzed by western blot using acetylated α-tubulin, cathepsin K, or β-actin antibody. **F** The culture media from OCs of WT or *Parkin*^−/−^ mice were treated with PD98059 (PD) or IL-1β was collected and the Cathepsin K activity was measured. **G** BMMs from *Parkin*^−/−^ mice were transduced with Mock or parkin (parkin O/E) retrovirus and cultured IL-1β. Transduced *Parkin*^−/−^ BMMs were analyzed for acetylated α-tubulin, parkin, and cathepsin K expression by western blot. **H**, **I** Transduced *Parkin*^−/−^ BMMs were cultured on bone slices and incubated with IL-1β. **H** Cathepsin K activity was measured, and **I** the number of resorption pits was estimated. *P < 0.05, **P < 0.005, and ***P < 0.001 between the indicated groups. Data are represented as means ± SD from three independent experiments; *P*-values were calculated by Kruskal–Wallis or Tukey post hoc comparison tests. **J** Schematic diagram of the proposed mechanism by which parkin regulates bone homeostasis

To provide the mechanistic explanation for aggravated inflammatory bone loss by parkin deficiency, we next explored the functional association of parkin with IL-1β-mediated signaling in OCs. IL-1β stimulation in parkin-depleted OCs compared to WT OCs significantly reinforced phosphorylation of ERK and acetylation of α-tubulin and was accompanied by HDAC6 reduction (Fig. 7A). Additionally, in line with the IL-1β-mediated increase in the number of TRAP-positive MNCs with actin rings and the area of TRAP-positive MNCs in the absence of parkin (Additional file 1: Fig. S3A−C), *Parkin*^−/−^ cells treated with IL-1β increased the number of resorption pits in dentin slices (Fig. 7B). Furthermore, in parallel with the augmented acetylation of tubulin, cathepsin K, an OC resorptive marker protein, was enhanced in IL-1β-treated *Parkin*^−/−^ cells relative to IL-1β-treated WT cells (Fig. 7C). Parkin depletion increased IL-1β-induced ERK activation accompanied by a reduction in the interaction with endogenous HDAC6 and tubulin (Fig. 7D). Moreover, the acetylated α-tubulin level was intensively increased by the IL-1β response in parkin-deficient cells, and this increase was ameliorated by an ERK inhibitor (Fig. 7E). IL-1β also enhanced the enzymatic activity of cathepsin K in the conditioned medium from OCs from both WT and *Parkin*^−/−^ mice (Fig. 7F). The blockade of this IL-1β-induced cathepsin K activity by a pERK inhibitor (PD98059) (Fig. 7E, F) suggests that IL-1β-induced parkin-deficient OCs have an increased bone-resorptive capacity that is mediated through pERK signaling.

Next, to examine whether ectopic parkin expression could reverse the effects of IL-1β on OC activity, we employed a retroviral system to overexpress parkin in *Parkin*^−/−^ BMMs. The expression levels of acetylated tubulin and cathepsin K were selectively restrained by parkin overexpression upon IL-1β stimulation (Fig. 7G). Furthermore, parkin overexpression in the homozygous deficient osteoclast precursor (OCP) cells decreased the number of resorption pits induced by IL-1β in mature OCs in association with inhibition of cathepsin K activity (Fig. 7H, I).

## Discussion

Decreased expression-associated polymorphisms in the regulatory region of parkin are linked to increased susceptibility to intracellular responses, such as cell death caused by oxidative stress, bacterial/viral infection, lipid metabolism, and tumor growth/metabolism [16, 17, 50–53]. Consistent with this, our current study showed an increased bone loss susceptibility upon *Parkin* gene dysfunction, providing new insights into the novel functions of parkin in bone pathology and thus broadening the scope of parkin functions beyond mitochondrial homeostasis.

Our results revealed that parkin dysfunction enhances bone-resorbing activity and subsequently induces bone loss in mice (Figs. 1 and 2), thus identifying parkin as a putative regulator of OCs. Notably, we found that acetylated tubulin is markedly increased in the podosome belt of mature OCs by the dysregulation of parkin (Fig. 3), and the “ERK-acetylated tubulin-cathepsin K” axis is a potential component modulator of the resorbing activity of OCs downstream of the parkin pathway (Fig. 7). In parallel, *Parkin*-deficient mice have exhibited tubulin hyper-acetylation in the corpus striatum during neuronal aging [40]. Of note in this regard, acetylated tubulin is regulated by the activity of the tubulin deacetylase HDAC6 via mDia2, which is an effector of Rho GTPase [34]. In the present study, the acetylated α-tubulin was enhanced in the RING domain-deficient mutant of parkin-transfected RAW 264.7 cells (Fig. 4C). These findings suggest that parkin could limit the acetylation of α-tubulin through parkin-HDAC6 interaction by decreasing RANKL-induced ERK activation in OCs, providing the mechanistic explanation for the direct role of parkin in regulating OC activation, as illustrated in Fig. 7J.

ERK activation by IL-1β stimulation is thought to be responsible for the enhancement of cathepsin K release by activating the Ca^2+^-dependent signaling pathway [54]. Here, we found that the increase in dentin resorption induced by IL-1β-induced ERK activation was more potent in *Parkin*^−/−^ OCPs than in WT OCPs (Fig. 7A–F) and significantly restricted by the ectopic expression of parkin (Fig. 7G–I), indicating that parkin deficiency reinforces IL-1β-mediated signaling for OC activation through ERK activation. These results provide evidence in support of a role for parkin in the bone-resorbing OC activity in the inflammatory condition. Together, IL-1β also reduces parkin expression (Additional file 1: Fig. S2), indicating that the level of parkin in OCPs may affect inflammatory bone erosion. Lipopolysaccharide (LPS) and TNF-α-dependent NF-κB signaling, which activate microglia cells and primary macrophages, also repress the parkin levels and thereby contribute to heightened inflammation-related gene expression [48]. In this line, inflammatory cytokines, such as TNF-α and IL-1β, may trigger both inflammation and bone destruction by suppressing parkin expression in OCPs.

Conversely, parkin deficiency amplifies antiviral inflammation by enhancing mitochondrial reactive oxygen species (mtROS) production to activate the NLRP3 inflammasome in macrophages [55], implying a possible pathogenic link between PD and the inflammatory response in macrophages. We showed from our current analysis that *Parkin*-deficient mice exhibit a more severe phenotype in terms of inflammation-mediated bone erosion together with an increase in bone destruction compared with WT mice (Fig. 6). Given that parkin deficiency leads to inflammation by upregulating IL-6, TNF-α, or IL-1β in mice [56], it is reasonable that parkin deficiency may sensitize mice to inflammation-induced bone erosion, as shown in this study (Fig. 6). However, in contrast to our present results, parkin deficiency reduces collagen antibody-induced arthritis (CAIA) [57]. Another study shows that *Parkin*-deficient mice are protected from LPS-induced lung injury due to lower pulmonary inflammation after LPS treatment [58]. Conversely, *Parkin*-deficient mice show increased inflammation, augmented innate antiviral inflammation, and a strong inflammatory phenotype following exhaustive exercise [55, 59, 60]. It is likely, however, that the high dose of LPS used in the CAIA and LPS-induced lung injury models, in contrast to other studies, including our current investigation, may affect the status of the inflammatory response in *Parkin*-deficient mice. Given that RA is associated with increased inflammation, it is feasible that parkin could be attenuated by feedback inhibition during inflammatory conditions and may play a crucial role in inflammation-mediated bone loss in RA. These literature findings suggest that parkin may act as a regulator of microtubule dysfunction, which can contribute to both PD and bone destruction in RA, providing the mechanistic explanation to support the pathologic link between PD and RA. Further studies are required to identify novel proteins that may interact with parkin to regulate OC activity.

## Conclusion

Parkin attenuates ERK-dependent acetylation of α-tubulin induced by RANKL through binding with HDAC6. These signaling pathways affect the stabilization of microtubules required for podosome formation to hinder excessive OC activity, thereby balancing OC activation for maintaining normal bone homeostasis. Under the inflammatory condition, IL-1β decreases parkin expression, which leads to an enhancement of ERK-dependent acetylation of α-tubulin and subsequent bone-resorbing capacity, which may cause deterioration in inflammatory bone erosion. This underlying mechanism suggests that parkin acts as a regulator of OC functions and inflammation-induced bone erosion.

## Materials and methods

### Mice and cell cultures

Male *Parkin*^−/−^ mice (006582: B6.129S4-Park2tm1Shn/J) with a C57BL/6 background were obtained from Jackson Laboratory. Experimental groups of male littermate mice were generated, genotyped, and housed. In brief, homozygous parkin knockout mice brought into the C57BL/6 genetic background by an accelerated backcross procedure were bred with female C57BL/6 mice. Mice of the heterozygous generation were then crossed to generate littermates of the six common genotypes by genotyping, as reported elsewhere [61]. Age-matched littermates of the genotypes of interest were used for all subsequent analyses. BMMs were isolated from the bones of these animals, as described elsewhere [62], and cultured in α-MEM (Cytiva) containing 10% heat-inactivated fetal bovine serum in the presence of M-CSF (30 ng/ml; Pepro Tech, Inc.). All animal experiments were approved by the Institutional Animal Care Committee of the Asan Institutes for Life Sciences (Seoul, Korea) (2019-13-199).

### Bone analyses

Distal femoral bones dissected from mice were fixed in 4% paraformaldehyde and scanned by micro-CT using the Skyscan 1072 system (14.85 μm pixel size, 50 kVp, 200 µA, 0.5 mm AI filter). Datasets were reconstructed using modified cone beam reconstruction software (NRecon) with a Feldkamp-based algorithm and segmented into binary images using adaptive thresholding. After the acquisition of 200 tomographic slices, a bone volume analysis was performed using CTan software (ver. 1.6). Three-dimensional surface-rendered models were generated using CTan software and visualized using CTVol (Bruker-micro-CT).

### BMD measurement for OVX-induced bone loss analysis

For BMD analysis in an ovariectomized mouse model, 8-week-old female WT and *Parkin*^−/−^ mice (*n* = 5 per group) were sham-operated or ovariectomized under anesthesia, and bone loss was assessed 4 weeks later. We extracted the femurs from euthanized mice, fixed them in 4% paraformaldehyde, and analyzed them by micro-CT (Skyscan), as described above.

### K/BxN serum transfer-induced arthritis model and histological analysis

Arthritic adult K/BxN mice were bled, and the sera were pooled. Recipient WT or *Parkin*^−/−^ mice were intraperitoneally injected on days 0 and 2. After injection, these animals were monitored daily until the end of the experiments (13 days). To evaluate arthritis severity, the following scoring system was employed: 0, no evidence of erythema or swelling; 1, erythema and mild swelling confined to the midfoot (tarsals) or ankle joint; 2, erythema and mild swelling extending from the ankle to the midfoot; 3, erythema and moderate swelling extending from the ankle to the metatarsal joints; or 4, erythema and severe swelling encompassing the ankle, foot and digits. The combined limb total score was recorded daily (maximum score, 16). The ankle thickness (mm) was measured with a caliper (Manostat).

### Osteoblast differentiation

Primary mouse osteoblast (OB) precursor cells were isolated from the calvariae of 1-day-old mice by six routine sequential digestions with 0.1% collagenase (Gibco BRL) and 0.2% dispase (Roche). The cells were then seeded onto 48-well culture plates at a density of 2 × 10^4^ cells/well and cultured in osteogenic medium (OM) (α-minimum essential medium [α-MEM], 10% fetal bovine serum [FBS], 10 mM β-glycerophosphate, and 50 mg/ml ascorbic acid) for 1 to 4 weeks. OB differentiation and mineralization were assessed by detecting alkaline phosphatase (ALP) activity or by staining with alizarin red S (AR).

### Osteoclast (OC) differentiation

Bone marrow-derived macrophages (BMMs) were isolated from the hind limbs of 6-week-old female WT and *Parkin*^−/−^ mice and cultured with 30 ng/ml M-CSF in the presence of 10 ng/ml RANKL (R&D Systems) for 3 days (3 × 10^4^ cells/15.6 mm dish). The cells were then fixed, stained for tartrate-resistant acid phosphatase (TRAP, Sigma-Aldrich) in accordance with the manufacturer’s instructions, and observed under a light microscope. OCs were identified as TRAP-positive multinucleated cells (TRAP^+^ MNCs; ≥ 3 nuclei). The number of TRAP^+^ MNCs in 48-well culture plates was counted using a Nikon ECLIPSE TS100 microscope and photographed using a Nikon DS-U3 camera. TRAP^+^ MNCs were counted by an investigator who was blind to the experiment. WT or *Parkin*^−/−^ osteoclast precursor cells (OCPs) primed by a low dose of RANKL for 24 h were further incubated in the presence of 10 ng/ml interleukin-1-beta (IL-1β) for OC generation (3 days), and the number of TRAP^+^ MNCs (≥ 3 nuclei) was evaluated, as described above.

### Bone resorption assay

WT- or *Parkin*^−/−^-derived macrophages (1 × 10^5^ cells) were cultured on dentine slices for 24 h in α-MEM medium containing 30 ng/ml M-CSF and 10 ng/ml RANKL and further incubated for 7 days. The medium was then removed, and 1 M NH_4_OH was added to the wells for 30 min. Adherent cells were removed from the dentine slices by ultrasonication; the resorption areas were visualized by staining with 1% toluidine blue. The resorbed pit area on the dentine was then photographed using an image analysis system (NIS-Elements Imaging Software) linked to a light microscope (Nikon). For inflammatory IL-1β-induced OC bone resorption, WT or *Parkin*^−/−^ OCPs primed with RANKL on a bone slice for 24 h were further incubated for 7 days in the presence of 10 ng/ml IL-1β, and the resorbed pit area sizes were evaluated, as described above. Resorption pit areas were quantified by ImageJ software.

### Western blot analysis

Western blot analysis of whole cell protein lysates was conducted. Briefly, differentiated mature osteoclasts (mOCs) at 3 days were collected and treated with lysis buffer (40 mM Tris-HCl, pH 8.0; 120 mM NaCl, 0.1% Nonidet-P40, 100 mM phenylmethylsulfonyl fluoride, 1 mM sodium orthovanadate, 2 µg/ml leupeptin, 2 µg/ml aprotinin). The extracted proteins were separated by sodium dodecyl sulfate-polyacrylamide gel electrophoresis (SDS-PAGE) and transferred to a nitrocellulose membrane. The membrane was blocked with 5% nonfat dry milk in Tris-buffered saline and incubated with primary antibodies against parkin and β-actin (Abcam), acetylated α-tubulin (Novus Biologicals), cathepsin K, ERK, p-ERK, p38, p-p38, IκB-α, FLAG, HDAC6, and α-/β-tubulin (Cell Signaling Technology) at 4 °C overnight. Blots were then incubated with a peroxidase-conjugated secondary antibody, and proteins were visualized by enhanced chemiluminescence (Supersignal® West Femto Maximum Sensitivity Substrate Kit, Thermo Fisher Scientific). The band density was quantified using ImageJ software (Version 1.6; National Institutes of Health).

### Transient siRNA transfection

BMM cells were seeded into 6-well plates at a density of 3 × 10^5^ cells and transfected with siRNA against negative control siRNA, parkin (GE Dharmacon), or HDAC6 (Santa Cruz Biotechnology) using Lipofectamine™ RNAiMAX (Thermo Fisher Scientific), according to the manufacturer’s instructions. A successful knockdown was identified by parkin or HDAC6 expression, as revealed by western blot analysis.

### Immunoprecipitation

Cells were lysed on ice for 20 min in a buffer containing 1% Triton X-100, 10 mM Tris (pH 7.6), 50 mM NaCl, 30 mM sodium pyrophosphate, 50 mM NaF, 5 mM EDTA, protease inhibitor cocktail (Roche), and phosphatase inhibitor cocktail (Roche). The resulting lysates were centrifuged at 16,000 × g at 4 °C, and the supernatant fraction was incubated with antibodies against FLAG (Cell Signaling Technology), and HDAC6 (Cell Signaling Technology) for 2 h at 4 °C, followed by incubation with Protein G Plus Agarose (Merck Millipore). Immunoprecipitants were washed two times with the lysis buffer, boiled in 5× SDS loading buffer for 10 min, followed by separation on SDS-PAGE and analysis by western blot. Western blot analysis was performed as described above. For inflammatory IL-1β-induced protein interaction, WT or *Parkin*^−/−^ OCPs were cultured for 3 days with 10 ng/ml of IL-1β in the presence of RANKL and M-CSF. Then, the anti-HDAC6 immunoprecipitation was performed.

### Retroviral gene transduction for parkin expression

Retroviral vector (pMXs-IRES-EGFP or pMXs-IRES-parkin-EGFP) was transfected into the packaging cell line, Plat E, using Lipofectamine 2000 (Thermo Fisher Scientific) in accordance with the manufacturer’s protocol. Viral supernatants were collected from the culture medium at 48 h after transfection. Calvarial osteoblastic precursor cells or BMMs from WT or *Parkin*^−/−^ mice were incubated with viral supernatants for 6 h in the presence of 8 µg/ml polybrene (Sigma-Aldrich). For further investigation, BMMs from *Parkin*^−/−^ mice transduced with pMXs-IRES-EGFP (Mock) or parkin (parkin overexpression) retrovirus were cultured with M-CSF and RANKL for 24 h, and then further incubated in the presence of 10 ng/ml IL-1β for 3 days.

### Immunofluorescence staining

OC precursors (3 × 10^4^ cells) were grown on coverslips in 48-well plates and differentiated into mOCs in medium containing M-CSF and RANKL for 3 days. Mature OCs were then washed twice with ice-cold phosphate-buffered saline (PBS) and fixed with 4% paraformaldehyde. After blocking with 1% bovine serum albumin in PBS for 30 min, cells were incubated with a primary antibody against parkin, actin, or tubulin for 24 h at 4 °C. Cells were then washed three times with PBS and incubated with a phalloidin probe (for F-actin; Molecular Probes), and then Cy™3- and DyLight™ 649-conjugated secondary antibodies for β-tubulin or acetylated α-tubulin (Molecular Probes), respectively, and FITC-conjugated secondary antibody for parkin (1:200 dilution; Molecular Probes). Nuclei were counterstained with 1 µg/ml DAPI. All images were obtained using a confocal laser scanning microscope (Carl Zeiss).

### CTX-1 measurements

Mouse plasma CTX-1 concentrations were measured using CTX-1-specific sandwich ELISA kits (Roche Diagnostics) in accordance with the manufacturer’s protocols. All samples were examined in triplicate for each experiment.

### Cathepsin K activity assay

The protease activity of cathepsin K from cultured media of OCs was measured using a Cathepsin K Activity Assay Kit (Abcam) in accordance with the manufacturer’s protocol.

### Plasmid construction for multiple domains of parkin and transfection

The full-length GFP-HDAC6 was purchased from OriGene Technologies, and p3xFLAG-parkin WT (FLAG-parkin) plasmid was a gift from Dong Hyun Sohn (Pusan University, Korea). Four multiple domains of parkin were constructed by inserting the cDNA into the p3xFLAG-CMV10 vector. Briefly, the FLAG-parkin and its deletion mutants (1–3) were constructed by inserting cDNA into the p3×FLAG-CMV10 vector (Sigma) between EcoRI and EcoRV sites. The multiple domain site mutants (Mt1, Mt2, or Mt3) of parkin were generated using the full-length FLAG-parkin plasmid as the template (see Additional file 1: Table S1 for detailed information). For cell transfection of plasmids, the transfection mixture used to transiently transfect the described cell lines was based on a mixture of plasmid DNA and Lipofectamine 3000 transfection reagent, as described in the procedures (Thermo Fisher Scientific). Plasmid DNA and transfection reagent were dissolved in α-MEM (without additives) and incubated at room temperature for 30 min. The mixture was added dropwise into the cell culture dish and incubated for 24 h in HEK 293 cells or further cultured for 3 days (including M-CSF and RANKL) in RAW 264.7 cells at 37 °C under an atmosphere of 5% CO_2_.

### Statistical analysis

All quantitative results were obtained from at least three independent experiments, and the data are represented as the means ± standard deviation. Post hoc or Kruskal–Wallis tests were performed using GraphPad Prism VI software (GraphPad Software, Inc.). Tukey post hoc tests were performed for multiple group comparisons. For two-group mean comparisons, student’s t tests were used. Values of *P* < 0.05 were considered statistically significant. *P* < 0.05, *P* < 0.005, or *P* < 0.001 are designated by 1, 2 and 3 asterisks, respectively.

## Supplementary Information

**Additional file 1: Figure S1.** Parkin expression in mOCs was colocalized with tubulin but not actin. mOCs from WT mice grown on glass coverslips were stained with phalloidin probe (for F-actin; red), Cy™3-conjugated antibodies (for β-tubulin; yellow), or/and FITC-conjugated antibodies (for parkin; green), counterstained for nuclei (DAPI, blue), and observed by confocal microscopy. **Figure S2.** Inflammatory cytokines reduce parkin expression. (A, B) BMMs (A) andRANKL-primed OCs (B) were treated with inflammatory cytokines, such as TNF-α (10 ng/ml) or IL-1β (10 ng/ml) for 48 h, and parkin protein expression was analyzed by western blot analysis. Densitometric quantification of parkin was compared to β-actin using ImageJ software. Data are represented as means ± SD. *P < 0.005, **P < 0.005, and ***P < 0.001 vs. buffer control. **Figure S3.** Depletion of parkin enhances IL-1β-induced OC activity. (A–C) Depletion of parkin does not affect differentiation but increases OC activity. BMMs from WT or *Parkin*^-/-^ mice in osteoclastogenic media were treated with IL-1β, fixed, and TRAP-stained. The (A) numbers of TRAP^+^ MNCs (≥ 3 nuclei), (B) TRAP^+^ MNCs with actin rings, and (C) the area of TRAP^+^ MNCs werequantified under a light microscope. Data are the mean ± SD; *P < 0.05, **P < 0.005, and ***P < 0.001 between the indicated groups. *P*-values were calculated by Tukey’s test for multiple comparisons. **Table S1.** Primer information for cloning of parkin-deletion mutants.

## Data Availability

The datasets used and/or analyzed during the current study are available from the corresponding author on reasonable request.

## Abbreviations

OC: Osteoclast
WT: Wild-type
*Parkin*^*−/−*^ OCP: Parkin-depleted-osteoclast precursor cell
HDAC6: Histone deacetylase 6
PD: Parkinson’s disease
BMD: Bone mineral density
IL-1β: Interleukin-1-beta
OC: Osteoclast
ERK: Extracellular signal-regulated kinase
RANKL: Receptor activator of nuclear factor kappa-B ligand
RA: Rheumatoid arthritis
TNF: Tumor necrosis factor
IL-6: Interleukin-6
OB: Osteoblast
OM: Osteogenic medium
FBS: Fetal bovine serum
ALP: Alkaline phosphatase
AR: Alizarin red S
BMM: Bone marrow-derived macrophage
TRAP: Tartrate-resistant acid phosphatase
TRAP^+^ MNC: TRAP-positive multinucleated cell
OCP: Osteoclast precursor cell
mOC: Mature osteoclast
SDS-PAGE: Sulfate-polyacrylamide gel electrophoresis
PBS: Phosphate-buffered saline
FLAG-parkin: p3xFLAG-parkin WT
qPCR: Quantitative PCR
*Parkin*^*−/−*^: *Parkin*-deficient
BV/TV: Bone volume per tissue volume
Tb. Th.: Trabecular thickness
Tb. N.: Trabecular number
Tb. Sp.: Trabecular separation
SMI: Structure model index
CTX-1: C-telopeptide of collagen type 1
M-CSF: Macrophage colony-stimulating factor
NF-κB: Nuclear factor kappa B
MAPK: Mitogen-activated protein kinase
pERK: Phosphorylated ERK
JNK: c-Hun N-terminal kinase
OVX: Ovariectomy
micro-CT: Micro-computed tomography
LPS: Lipopolysaccharide
mtROS: Mitochondrial reactive oxygen species
CAIA: Collagen antibody-induced arthritis
Ac α-tubulin: Acetylated α-tubulin
H/E: Hematoxylin/eosin
