# Supplementary material for: Dysfunction in parkin aggravates inflammatory bone erosion by reinforcing osteoclast activity
Source: Cell Biosci. 2023 Mar 7;13:48. doi: 10.1186/s13578-023-00973-0 (PMC9993703; doi:10.1186/s13578-023-00973-0)
Supplement: Supplementary file 1 — Additional file 1: Figure S1. Parkin expression in mOCs was colocalized with tubulin but not actin. mOCs from WT mice grown on glass coverslips were stained with phalloidin probe (for F-actin; red), Cy™3-conjugated antibodies (for β-tubulin; yellow), or/and FITC-conjugated antibodies (for parkin; green), counterstained for nuclei (DAPI, blue), and observed by confocal microscopy. Figure S2. Inflammatory cytokines reduce parkin expression. (A, B) BMMs (A) andRANKL-primed OCs (B) were treated with inflammatory cytokines, such as TNF-α (10 ng/ml) or IL-1β (10 ng/ml) for 48 h, and parkin protein expression was analyzed by western blot analysis. Densitometric quantification of parkin was compared to β-actin using ImageJ software. Data are represented as means ± SD. *P < 0.005, **P < 0.005, and ***P < 0.001 vs. buffer control. Figure S3. Depletion of parkin enhances IL-1β-induced OC activity. (A–C) Depletion of parkin does not affect differentiation but increases OC activity. BMMs from WT or Parkin-/- mice in osteoclastogenic media were treated with IL-1β, fixed, and TRAP-stained. The (A) numbers of TRAP+ MNCs (≥ 3 nuclei), (B) TRAP+ MNCs with actin rings, and (C) the area of TRAP+ MNCs werequantified under a light microscope. Data are the mean ± SD; *P < 0.05, **P < 0.005, and ***P < 0.001 between the indicated groups. P-values were calculated by Tukey’s test for multiple comparisons. Table S1. Primer information for cloning of parkin-deletion mutants. [file 13578_2023_973_MOESM1_ESM.docx]

## Additional file 1

##
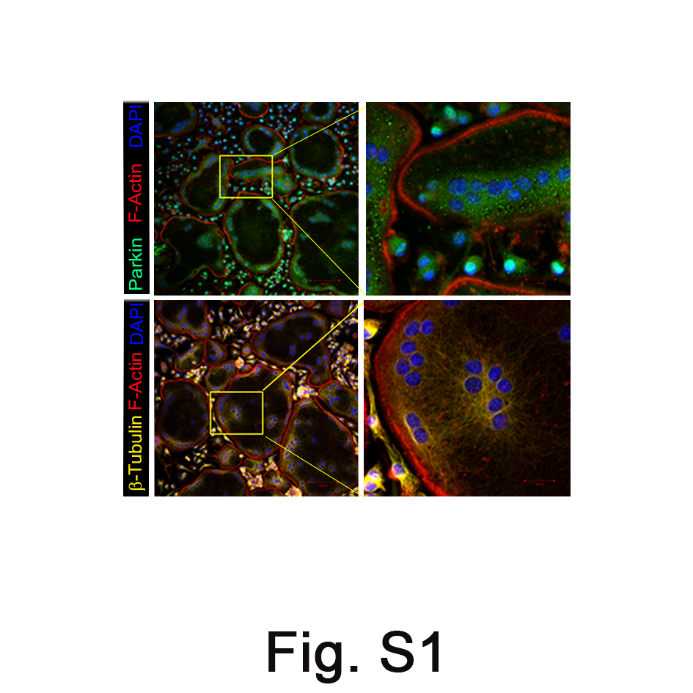


**Fig. S1. Parkin expression in mOCs was colocalized with tubulin but not actin.** mOCs from WT mice grown on glass coverslips were stained with phalloidin probe (for F-actin; red), Cy™3-conjugated antibodies (for β-tubulin; yellow), or/and FITC-conjugated antibodies (for parkin; green), counterstained for nuclei (DAPI, blue), and observed by confocal microscopy.


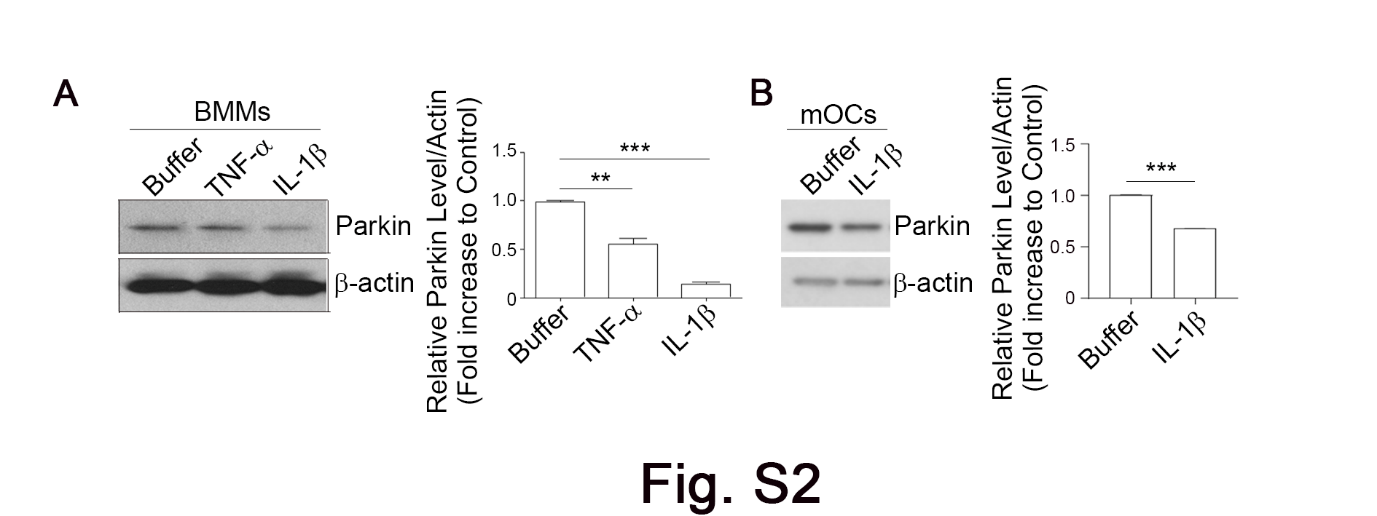


**Fig. S2. Inflammatory cytokines reduce parkin expression.** (A, B) BMMs (A) and RANKL-primed OCs (B) were treated with inflammatory cytokines, such as TNF-α (10 ng/ml) or IL-1β (10 ng/ml) for 48 h, and parkin protein expression was analyzed by western blot analysis. Densitometric quantification of parkin was compared to β-actin using ImageJ software. Data are represented as means ± SD. **P* < 0.005, ***P* < 0.005, and ****P* < 0.001 vs. buffer control.

**
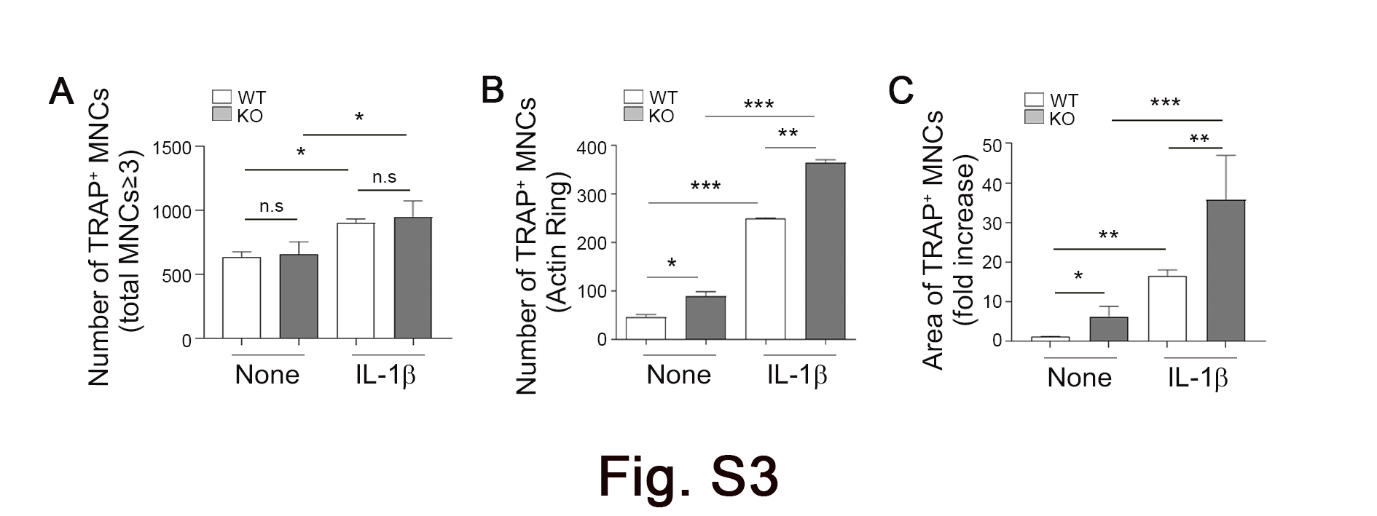
**

**Fig. S3. Depletion of parkin enhances IL-1β-induced OC activity.** (A-C) Depletion of parkin does not affect differentiation but increases OC activity. BMMs from WT or *Parkin*^-/-^ mice in osteoclastogenic media were treated with IL-1β, fixed, and TRAP-stained. The (A) numbers of TRAP^+^ MNCs (≥3 nuclei), (B) TRAP^+^ MNCs with actin rings, and (C) the area of TRAP^+^ MNCs were quantified under a light microscope. Data are the mean ± SD; **P* < 0.05, ***P* < 0.005, and ****P* < 0.001 between the indicated groups. *P*-values were calculated by Tukey's test for multiple comparisons.

**Table S1.** Primer information for cloning of parkin-deletion mutants.

| Gene | Oligo | | Amplicon size (bp) |
| --- | --- | --- | --- |
| Mt1 of *parkin* | Parkin_*Eco*R1-F | 5'-TTGCGGCCGCGAATTCAatgatagtgtttgtcaggttcaactcc-3' | 672 |
|  | Mt1_*Eco*RV-R | 5'-TCGACTGGTACCGATATCctatgaggttgggtgtgctcc-3' |  |
| Mt2 of *parkin* | Parkin_*Eco*R1-F | 5'-TTGCGGCCGCGAATTCAatgatagtgtttgtcaggttcaactcc-3' | 951 |
|  | Mt2_*Eco*RV-R | 5'-TCGACTGGTACCGATATCctactgctcttctccaaggatcctg-3' |  |
| Mt3 of *parkin* | Parkin_*Eco*R1-F | 5'-TTGCGGCCGCGAATTCAatgatagtgtttgtcaggttcaactcc-3' | 1236 |
|  | Mt4_*Eco*RV-R | 5'-TCGACTGGTACCGATATCctaggcctcctcccagcg-3' |  |
